# Evaluation of the Project P.A.T.H.S. Based on Students' Weekly Diaries: Findings from Eight Datasets

**DOI:** 10.1100/2012/354254

**Published:** 2012-04-19

**Authors:** Daniel T. L. Shek, Rachel C. F. Sun

**Affiliations:** ^1^Department of Applied Social Sciences, The Hong Kong Polytechnic University Kowloon, Hong Kong; ^2^Public Policy Research Institute, The Hong Kong Polytechnic University Kowloon, Hong Kong; ^3^Department of Social Work, East China Normal University, Shanghai, China; ^4^Kiang Wu Nursing College of Macau, Macau; ^5^Division of Adolescent Medicine, Department of Pediatrics, Kentucky Children's Hospital, University of Kentucky College of Medicine, Lexington, KY 40506-9983, USA; ^6^Faculty of Education, The University of Hong Kong Pokfulam, Hong Kong

## Abstract

This paper aims to investigate the effectiveness of the Tier 1 Program of the Project P.A.T.H.S. (positive adolescent training through holistic social programmes) based on eight datasets collected between 2005 and 2009. A total of 1,138 students who participated in the program were randomly invited (from the whole grade or in some classes) to write a piece of journal in the form of a weekly diary in order to reveal their perceptions and feelings regarding the program and the perceived benefits of the program. Based on an integration of findings from different databases, results showed that the respondents generally (1) had positive views on the program, (2) had positive views on the instructors, and (3) perceived that they had acquired competencies at the societal, school, familial, interpersonal, and personal levels after joining the program. Acknowledging the limitations of diaries, the present qualitative findings provide support for the effectiveness of the Tier 1 Program of the Project P.A.T.H.S. in Hong Kong.

## 1. Introduction

The purpose of this paper is to report evaluation findings of a positive youth development program in Hong Kong (Project P.A.T.H.S., i.e., positive adolescent training through holistic social programmes) based on weekly diaries written by students participating in the program. The personal diary is an important source of information to understand the social reality. For example, Anne Frank's diary documents the thoughts, feelings, and experiences of a Jewish teenage girl who had fled and hid in Amsterdam with her family when Nazi forces conquered The Netherlands. In view of the invaluable information gathered from people's diaries, diaries for analyzing human behavior, discovering history, or evaluating a program represent an attractive research approach. In the context of social sciences research, diaries are commonly used in nonexperimental research to collect data on human behavior. For example, Leigh [1] asked participants to record their time, quantity, and type of drinking and sexual activity over 10 weeks in order to examine the temporal relationships of alcohol consumption to sexual behavior. Behavioral diaries have also been used in behavioral modification programs [2]. There are studies to suggest the strengths and weaknesses of using daily diaries as a research strategy [3, 4], and the debate has continued in the literature [5, 6].

In the field of education, it is not uncommon to find that a teacher would keep reflective journals as a means to assess personal teaching performance, identify one's pedagogical strengths and weaknesses, and evaluate the effectiveness of the changes implemented in teaching [7]. It is believed that a reflective journal “suggests question, identifies new areas to explore, reveals meaningful absences, and uncovers recurring patterns” ([8], page 16). Therefore, some studies utilized teachers' reflective diaries as a kind of data collection method to investigate the essential components of effective teaching practice or pedagogy, and to examine ways of contributing to professional development [9].

Similarly, in contrast to the traditional learning model where students mainly take a passive role in receiving what their teachers' transmit or reciting the information from books, students are perceived as active learners who construct their personal knowledge based on their own experiences in reflective learning models, where they are encouraged to be autonomous learners who reflect on their learning and to have more awareness on their learning processes, competencies, and limitations. Therefore, reflective observation is highlighted to be an indispensable process in translating concrete experiences to concepts and actions in the experiential learning process [10], in which keeping a diary is considered as an opportunity for personal reflection that, in turn, enhances students' autonomy and develops an ownership of their learning process [11].

In order to guide students to become autonomous learners, students studying in human services programs were commonly asked to write diaries or reflective journals in different ways. First, some courses simply aimed to encourage students to be critical and reflective learners. For example, in a module on management education, students were asked to write eight diaries with predesigned topics throughout the course [12]. Second, some courses were targeted towards strengthening the students' ability to integrate their course learning into actual field settings. For example, social work students were required to write reflective journals with predesigned questions at several stages of their field work, on top of other written assignments [13]. Third, students were guided to be determinant masters of their future. For example, students in a health-discipline course were asked to write a journal with no specific guidelines on their experiences of their practices in rural areas, so as to identify the positive and negative aspects of rural practices and develop their interest in working in the rural areas in the future [14]. Chaloner [15] affirmed that “using a personal journal implies that the learner will construct a memory of the learning experience and how it can be applied to the workplace by using a personal narrative. Through such an individualized and personal approach, it is hoped that the learner will be motivated enough to assume the autonomy that he/she is being offered” (page 22).

Besides understanding the subjective world views of the informants, a survey of the literature in the field of evaluation shows that there is an increasing number of studies using diaries as a tool for evaluation. Wagner [16] used student journals to evaluate the effectiveness of a course. Follick et al. [17] evaluated a daily activity diary for chronic pain patients. Travers [18] examined the use of a reflective diary methodology for exploring the lived experiences of stress and coping among 30 college students. Cohen et al. [19] examined the development and use of an online diary in the evaluation of a preventive care program that provided real-time communication between the evaluator and participants. Knowles and Tarrier [20] evaluated diary intervention on levels of anxiety and depression in a group of intensive care unit survivors and demonstrated the therapeutic effects of diaries.

The main purpose of this paper is to report evaluation findings of the Project P.A.T.H.S. using student diaries as an evaluation method. The project P.A.T.H.S. is a positive youth development program that attempts to promote holistic development among adolescents in Hong Kong, with an initial earmarked grant of HK$400 million by The Hong Kong Jockey Club Charities Trust. The Project is divided into two tiers of programs. The Tier 1 Program is a universal curriculum-based program for secondary 1 to 3 students in participating schools and the Tier 2 Program is a selective program for students with greater psychosocial needs. In contrast to mainstream approaches that focus on tackling youth developmental problems, the field of positive youth development focuses on enabling the talents, strengths, interests, and future potentials in children and adolescents. Roth et al. [21] pointed out that positive youth development programs are commonly regarded as “programs that provide opportunities and support to help youth gain the competencies and knowledge they need to meet the increasing challenges they will face as they mature” (page 423). Although many western programs have been developed to promote positive development in children and adolescents, not all of them are successful in promoting adolescent development. For example, Catalano et al. [22] reviewed 77 programs on positive youth development, and their review showed that only 25 programs were successful. Obviously, evaluation is an important issue to be considered when assessing the effectiveness of positive youth development programs. 

To understand the program effects of the Project P.A.T.H.S., several complementary program evaluation strategies, including both quantitative and qualitative methodologies, have been adopted [23]. These included objective outcome evaluation, subjective outcome evaluation, process evaluation, interim evaluation, and qualitative evaluation based on focus groups, in-depth interviews, and case studies. Through the adoption of different evaluation strategies, there is multiple evidence supporting the effectiveness of the Tier 1 Program of the Project P.A.T.H.S. based on different types of data collected from different participants utilizing different methods. For example, both quantitative findings [24, 25] and qualitative findings [26, 27] based on different studies consistently suggest that the Project P.A.T.H.S. is helpful in promoting the psychosocial competencies of the program participants.

To further understand the perceived program effects of the Tier 1 Program, evaluation findings based on weekly diaries written by students are reported in this paper. Actually, there are published papers on the evaluation of the Project P.A.T.H.S. based on weekly diaries [28, 29]. In this paper, the findings based on weekly diaries in eight datasets were aggregated in order to give a picture regarding the effectiveness of the Project P.A.T.H.S. in Hong Kong. In short, the purpose of this paper is to give a descriptive picture on the views and experiences of the program participants.

## 2. Methods

### 2.1. Participants and Procedures

From 2005 to 2009, a total of 244 secondary schools participated in the P.A.T.H.S. Project. Among all the participating schools, 12 schools that joined the secondary 1 level of the Project, 12 schools that joined the secondary 2 level, and 10 schools that joined the secondary 3 level were randomly selected and invited to take part in eight research studies. In each of the selected schools, the school contact person was asked to randomly invite some students (from the whole grade or in some classes) to write a piece of journal in the form of a weekly diary in order to reveal their perceptions and feelings after joining the Tier 1 Program of the project. The students were informed of the purpose and confidentiality of this study, and their consent for participation was sought prior to data collection. The title of the reflective journal was “Participating in the Tier 1 Program of the Project P.A.T.H.S.: Experiences and Feelings.” The reflection was expected to be not less than 200 words in Chinese, and it should be related to the students' experiences, feelings, and comments in connection with their participation in the Tier 1 Program. The students could either complete it at home or during class time. The total number of students' diaries received was 1,138, comprising 369 written by secondary 1 students, 486 written by secondary 2 students, and 283 written by secondary 3 students (see Table 1). As the cases were randomly selected and the response rate was 100%, the recruited sample could be regarded as respectable. No secondary 1 students were recruited in the 2007/2008 school year because several cohorts of data had been collected.

While the basic research design of the studies generating the datasets was primarily qualitative in nature (i.e., written comments on the program, instructors, and benefits), it is possible to use quantitative methods (such as counting and frequency analyses) to create a descriptive profile of the findings [28]. In the present study, the secondary data analysis approach was adopted by integrating and synthesizing the data generated from the different databases. 

### 2.2. Data Analyses

In each of the eight studies, the data were analyzed using general qualitative analyses techniques involving three steps [30]. First, relevant raw codes were developed for words, phrases, and/or sentences that formed meaningful units at the raw responses level. Second, the codes were further combined to reflect higher-order attributes at the category of codes level (i.e., themes and subthemes). Third, the categories of codes were further analyzed to reveal the broader themes at the thematic level. Three main themes were identified from the weekly diaries: views on the program, views on the instructors, and perceived benefits of the program. Both intra- and interrater reliabilities on the coding regarding these views were calculated, and the results were satisfactory (85–100%). In the present analyses, because the researchers designed the program of the Project P.A.T.H.S., they were conscious of their own biases and expectations on the program to be effective. This paper reports the analyses based on the aggregated findings of these eight studies. The major categories and subcategories of responses are presented in the tables, and narrative illustrating the themes can be seen in the Section 3.

## 3. Results

Responses regarding the students' perception of the program and the instructor, as well as the perceived benefits of the program, are presented in this paper. The integrated findings are presented in Tables 2–4. As shown in Table 2, there was a total of 2,290 meaningful units regarding students' views on the Tier 1 Program implemented from 2005 to 2009. The responses were categorized into “overall impression,” “program content,” “learning process,” “program arrangement,” and “other comments.” About 81% of the total responses was coded as positive. Most of the respondents had positive views on the “overall impression,” followed by “learning process” and “program content”. There were 85.4, 77.2, and 83.1% of secondary 1, 2, and 3 students who reported positively on the program, respectively, although some negative comments were recorded. Three cases illustrating the respondents' perceptions of the program at each grade are presented below.

**Student A (Secondary 1, 2006/2007)**

“This is my first time joining the P.A.T.H.S. Project. I learned about emotion, choice and other knowledge about development from the program … I personally think that this program has great impact on me and my classmates. We could learn via the worksheet activities as well as from the advice given by the classmates. What we learned are closely related to reality which could be applicable to daily life. Therefore, I think the program is very meaningful.”

**Student B (Secondary 2, 2007/2008)**

“After completion of the Tier 1 Program in the first semester, I felt I became more mature in interpersonal communication and personal growth. We studied various problems such as teenagers' learning attitudes and hedonism during class. Through games or group discussion, we learned how to analyze and acquire correct information, and how to face difficulties and remain positive under adverse circumstances. I like this program very much because all the program content is closely related to common teenage problems, such as body shaping and substance abuse, which is closely related to our daily life. If we do not have good virtues and healthy mind, we will follow the wrong track easily. Therefore, the program serves as not only a light in the dark but also a teacher who teaches and guides us to the right way.”

**Student C (Secondary 3, 2008/2009)**

“Fortunately, I joined the Project P.A.T.H.S. from Secondary 1 to Secondary 3. This is one of my favorite subjects. The atmosphere during the class was relaxing that made the subject become more acceptable. Moreover, I really liked the design of the program. Teachers and social workers used multiple resources for teaching in order to guide the students to think from different angles and make judgment. Problems such as school orientation, conflicts with parents and peer relationship that troubled me a lot were discussed in the class. I could solve these problems by applying the skills I learned from the class. Obviously the project works.”

There were 558 responses regarding “views on instructors,” which were categorized into “overall impression,” “teaching performance,” “teaching attitude,” and “others” (see Table 3). About 90% was positive responses. Most of the respondents had positive views on instructors' “teaching performance.” Several cases illustrating the respondents' views on the instructors are shown below.

**Student D (Secondary 1, 2006/2007)**

“I remembered when I first joined the Project P.A.T.H.S., I felt very excited … Miss Wong was our instructor. Although the topics of each P.A.T.H.S. session were different, the goal was the same—to help students to develop good interpersonal relationship. The instructor would encourage the students to share their opinions on a topic and then asked other students to discuss or analyze each student's viewpoints. The instructor would also arrange some activities such as group discussion and role-play. These activities promoted the relationship among classmates in only one year's time.”

**Student E (Secondary 3, 2008/2009)**

“There was a period of time when I lost self-confidence due to a failure. I kept myself in the corner of my world and could not find a way out until I heard some sharing from the instructor. She said, “Life is like an obstacle race. No matter what obstacles you encounter during the race, once you pass the finishing line, you will have victory.” I suddenly realized that self-pitying did not help anything. Rather, I should regain my strength to face new challenges. The words from the instructor become my motto, which drives me to keep moving forward.”

The perceived benefits of the program to the students are shown in Table 4. The 2,986 meaningful units were categorized into six main categories (i.e., societal, school, familial, interpersonal, personal levels of competence, and others). Most of the responses were related to the effectiveness of the program on a personal level (*n* = 1,936), particularly in the subcategory of “positive self” (*n* = 378, e.g., “enhanced self-understanding”), followed by “general gains” (*n* = 312, e.g., “acquired skills/knowledge”) and “cherishing life” (*n* = 310, e.g., “promoted reflection of life”). In addition, many respondents reported that they acquired interpersonal competence (*n* = 773), which could be categorized into “general interpersonal competence” (e.g., “got along with others”) and “specific interpersonal competence” (e.g., “learned to accept, care, trust, respect, and understand others”). Some narratives based on the respondents revealed that the program was perceived positively by the program participants.

**Student F (Secondary 1, 2006/2007)**

“I learned much useful knowledge in this program. Firstly, I learned many interpersonal skills such as how to get along with classmates, teachers, friends, senior people and family members. I also learned to appreciate others and myself. I knew myself much more than before. By realizing my strengths and weaknesses, I was able to improve myself. In addition, I learned to distinguish between what I should do and what I should not. Most important of all, the program enabled me to meet new friends and improve my communication skills. All these skills will be very useful for my future development.”

**Student G (Secondary 2, 2007/2008)**

“When I looked back, I really learned a lot of things. I felt I truly grew up. In familial aspect, I understood that family members should mutually help each other and care for one another. I need to help doing the family chores when I have time. As parents have been taking care of us whole-heartedly, we have to repay and be thankful to them. I learned the importance of communication. Now I would spend more time to chat with my parents and share my feelings with them. In doing so, my parents were reassured and became less worried about me … In social aspect, I knew that we should follow social disciplines such as keeping the voice down in public area to avoid interrupting other people … In global aspect, I learned that there were many people in the world living in poverty. We should do whatever we could do to help these people.”

**Student H (Secondary 3, 2008-2009)**

“I could still remember when I first participated in the Project P.A.T.H.S., I did not understand why we should sacrifice two sessions of lesson for studying this program. However, I was gradually involved in it. This program provided me an opportunity to know more about my teachers and classmates … Although the three-year program is going to end, we still have a lot to learn throughout our lives. Among the units I have learned, the most impressive topic is “Life and Death”. “Life and Death” is actually not far away from us. Remember the Wenchuan earthquake last year? Life of the students in Sichuan was changed in only one second. As Hong Kong students, we should make deepest reflection—bliss is not taken for granted!”

## 4. Discussion

The purpose of this study was to evaluate the Tier 1 Program of the Project P.A.T.H.S. using findings based on weekly diaries collected in the experimental and full implementation phases (2005–2009) of the project. There are several characteristics of this study. First, a large sample of participants (*n* = 1,138 students) was involved. Second, eight datasets collected across three grades at different points in time were analyzed. Third, this is the first known scientific evaluation study of a positive youth development program utilizing student diaries based on different cohorts in China. Fourth, this is also the first evaluation study utilizing diaries based on such a large sample of participants in the global context. Finally, the data were analyzed in both quantitative and qualitative ways. As far as the design and data collection of the studies are concerned, the study has the characteristics of naturalistic inquiry, inductive analysis, holistic perspective, qualitative data, unique case orientation, and contextual sensitivity. Regarding data analyses, both qualitative (e.g., coding and categorizing) and quantitative (mainly counting) analyses were used.

Based on the weekly diaries written by the students, the studies reported in this paper showed that the participants generally had positive perceptions of the Tier 1 Program of the Project P.A.T.H.S. and the instructors. The findings presented in Tables 2 and 3 suggest that the participants had positive impressions of the program, and they appreciated the program content and learning process involved. In addition, Table 4 shows that the participants overwhelmingly regarded the program as beneficial to the cognitive, emotional, social, and productive quality of life of the program participants. For example, a sizable number of responses were related to positive self-image after joining the program (Table 4). In addition, many participants remarked that their interpersonal competencies were enhanced after joining the program. In short, the findings in Table 4 basically suggest that the program is beneficial to the development of the program participants.

The above findings are generally consistent with previous research findings in the experimental and full implementation phases, and they basically concur with both the quantitative and qualitative findings based on objective outcome, subjective outcome, qualitative, and process evaluation findings (e.g., [24–27, 31]). In short, the present findings provide additional evidence for supporting the effectiveness of the Tier 1 Program of the Project P.A.T.H.S. in Hong Kong. From the perspective of triangulation, the existing evaluation findings suggest that the effectiveness of the Tier 1 Program of the Project P.A.T.H.S. is supported by evaluation data collected from different sources and by different strategies.

The present findings demonstrate the utility of using student diaries to evaluate positive youth development programs. By asking the program participants to reflect on their experiences, subjective perceptions and perceived benefits of the program can be properly understood in a less mechanical and artificial manner. Such an approach can help to capture the dynamic nature of the perceived qualities and effectiveness of the program. In addition to its holistic emphasis and ease of data collection, Maxwell [32] commented that “qualitative research is a rigorous means of investigating causality” (page 3). Slayton and Llosa [33] also remarked that “qualitative methods can confirm that it is actually the program that is responsible for the effect” (page 2544). Obviously, the use of reflective journals enjoys the strengths of qualitative studies in assessing the changes in quality of life among the participants after joining the program.

Shek et al. [34] proposed that several principles should be maintained in order to enhance the quality of qualitative research. As a qualitative study, the following attributes are intrinsic to this study. First, a general qualitative orientation was adopted. Second, recruitment processes for the participants and justifications for the number of participants are described. Third, details of the data collection are given. Fourth, issues of biases and ideological preoccupation are addressed. Fifth, inter- and intrarater reliabilities information is presented. Finally, the categorized data are kept in a systematic filing system in order to ensure that the findings are auditable.

Nevertheless, several limitations of the study should be noted. First, for students who are sensitive about the issue of “invasion of privacy” [12], the use of a weekly diary may be regarded as an obtrusive evaluation device. Second, as the diary method is written in nature, students who are not good at using words to express their experiences may write very little. This problem may be particularly acute in schools that admit students with poor academic performance or literacy. This is also a problem in the Chinese culture where Chinese people may lack the language to describe their feelings. Third, as there was only one occasion on which the participants expressed their views in a one-way manner, it was not possible to have dialogues with the informants to further understand some of their views. In particular, theoretical sampling approaches could be attempted in future studies.

Fourth, although the present findings are interpreted in terms of the positive program effects and experiences of the program participants, it should be noted that there are several alternative explanations. These alternative explanations should be considered because high proportions of responses were positive across the eight studies. The first alternative explanation is that the students were afraid of being punished by the program implementers if they did not respond in a favorable manner. However, this possibility can be partially dismissed because the teachers did not mark the weekly diaries and the identities of the students were basically anonymous. The second alternative explanation is that the students consciously acted in a “nice” manner to help the workers to illustrate a positive program effect. However, this alternative explanation can be partially dismissed because negative comments were recorded and students are normally encouraged to express their views honestly. The third alternative explanation is that the high proportion of positive responses observed, were in fact, biased responses. However, this alternative explanation can also be dismissed because the schools and students were randomly selected. The fourth alternative explanation is that because there is no control group, natural developmental maturation may account for the positive evaluation findings. That is, through maturation, the participants may see things in a more favorable manner. Furthermore, threats to internal validity in the study should be realized.

Finally, although the 11 principles proposed by Shek et al. [34] were upheld in this study, peer and member checking (principle 8) was not carried out because of time and manpower constraints. Despite these limitations, this study provides pioneering evaluation findings across samples and time that support the positive nature of the Project P.A.T.H.S. and its effectiveness in promoting holistic youth development among Chinese adolescents in Hong Kong.

## Figures and Tables

**Table 1:** Number of respondents writing weekly diaries.

	S1			S2			S3	
	Study 1 2005/2006 EIP	Study 2 2006/2007 FIP	Study 3 2008/2009 FIP	Study 4 2006/2007 EIP	Study 5 2007/2008 FIP	Study 6 2008/2009 FIP	Study 7 2007/2008 EIP	Study 8 2008/2009 FIP
Total schools that joined P.A.T.H.S.	52	207	197	49	196	198	48	167
(i) 10-hour program	23	95	104	27	113	110	29	104
(ii) 20-hour program	29	112	93	22	83	88	19	63
Total schools that joined this study	4	6	2	4	6	2	4	6
(i) 10-hour program	1	0	0	0	0	0	0	0
(ii) 20-hour program	3	6	2	4	6	2	4	6
No. of diaries collected	95	216	58	260	164	62	132	151
Percentage of informants in the selected schools	15%	19%	15%	36%	15%	16%	24%	16%

Note: S1: secondary 1 level; S2: secondary 2 level; S3: secondary 3 level; EIP: experimental implementation phase; FIP: full implementation phase.

**Table 2:** Views on the program across study 1 to study 8.

Views on the program	Responses	S1			S2			S3		Total
		Study 1 2005/06 EIP	Study 2 2006/07 FIP	Study 3 2008/09 FIP	Study 4 2006/07 EIP	Study 5 2007/08 FIP	Study 6 2008/09 FIP	Study 7 2007/08 EIP	Study 8 2008/09 FIP	
Overall impression	Positive: (e.g., meaningful, excellent, beneficial, and liked the program)	26	116	43	274	37	50	59	106	711
	Negative: (e.g., meaningless, bad, disappointed, and not interesting)	0	1	5	18	9	7	2	8	50
	Neutral: (e.g., mixed blessings, fair, not different from other programs, and similar to the subjects in primary schools)	3	0	0	3	0	1	0	3	10

	Subtotal	29	117	48	295	46	58	61	117	771

Program content	Positive: (e.g., practical, creative, rich content, and easily understood)	27	25	35	68	31	43	78	66	373
	Negative: (e.g., disliked growth puzzles, boring content, too many worksheets, and dissatisfied with the activities)	6	30	10	8	26	49	24	21	174
	Neutral: (e.g., comments on teaching materials)	0	0	0	0	0	0	0	2	2

	Subtotal	33	55	45	76	57	92	102	89	549

Learning process	Positive: (e.g., funny, interesting, happy, and active participation)	34	70	25	107	62	26	48	102	474
	Negative: (e.g., boring, uninvolved, poor learning atmosphere, and nonsense)	5	18	2	96	0	3	33	3	160

	Subtotal	39	88	27	203	62	29	81	105	634

Program arrangement	Positive: (e.g., good delivery mode, good arrangement, and reward scheme)	0	0	5	0	1	10	0	26	42
	Negative: (e.g., inadequate resources, poor arrangement, insufficient time, and bad classroom management)	0	0	0	0	5	4	6	7	22

	Subtotal	0	0	5	0	6	14	6	33	64

Other comments	Positive	23	55	7	97	9	5	41	17	254
	Negative	0	4	0	6	0	0	0	1	11
	Neutral	0	0	0	7	0	0	0	0	7

	Subtotal	23	59	7	110	9	5	41	18	272

Grand total	Positive (%)	110 (88.7)	266 (83.4)	115 (87.1)	546 (79.8)	140 (77.8)	134 (67.7)	226 (77.7)	317 (87.6)	1854
	Negative (%)	11 (8.9)	53 (16.6)	17 (12.9)	128 (18.7)	40 (22.2)	63 (31.8)	65 (22.3)	40 (11)	417
	Neutral (%)	3 (2.4)	0	0	10 (1.5)	0	1 (0.5)	0	5 (1.4)	19

	Total (%)	124 (100)	319 (100)	132 (100)	684 (100)	180 (100)	198 (100)	291 (100)	362 (100)	2290

Total in each grade	Positive (%)	491 (85.4)			820 (77.2)			543 (83.1)		1854
	Negative (%)	81 (14.1)			231 (21.8)			105 (16.1)		417
	Neutral (%)	3 (0.5)			11 (1)			5 (0.8)		19

	Total (%)	575 (100)			1062 (100)			653 (100)		2290

**Table 3:** Views on the instructor across study 1 to study 8.

Views on the instructor	Responses	S1			S2			S3		Total
		Study 1 2005/2006 EIP	Study 2 2006/2007 FIP	Study 3 2008/2009 FIP	Study 4 2006/2007 EIP	Study 5 2007/2008 FIP	Study 6 2008/2009 FIP	Study 7 2007/2008 EIP	Study 8 2008/2009 FIP	
Overall impression	Positive: (e.g., nice, liked instructors, could motivate students, and thankful to instructors)	2	8	0	41	2	6	22	16	97
	Negative: (e.g., disgusting, strict, always blaming, and inconsistent facilitation skills between instructors)	3	1	0	3	0	0	0	0	7

	Subtotal	5	9	0	44	2	6	22	16	104

Teaching performance	Positive: (e.g., excellent, well prepared, professional, and shared own experiences)	9	28	17	51	28	17	27	45	222
	Negative: (e.g., boring teaching, poor classroom management, poor teaching skills, and taught in a hurry manner)	0	3	0	4	3	0	23	1	34

	Subtotal	9	31	17	55	31	17	50	46	256

Teaching attitude	Positive: (e.g., enthusiastic, devoted, willing/able to help students, and patient)	5	7	4	40	10	12	27	57	162

	Subtotal	5	7	4	40	10	12	27	57	162

Others	Positive	3	3	0	15	0	0	4	0	25
	Negative	0	0	0	3	0	0	2	0	5
	Neutral	0	0	2	2	0	2	0	0	6

	Subtotal	3	3	2	20	0	2	6	0	36

Grand total	Positive (%)	19 (86)	46 (92)	21 (91.3)	147 (92.4)	40 (93)	35 (94.6)	80 (76.2)	118 (99.2)	506
	Negative (%)	3 (14)	4 (8)	0	10 (6.3)	3 (7)	0	25 (23.8)	1 (0.8)	46
	Neutral (%)	0	0	2 (8.7)	2 (1.3)	0	2 (5.4)	0	0	6

	Total (%)	22 (100)	50 (100)	23 (100)	159 (100)	43 (100)	37 (100)	105 (100)	119 (100)	558

Total in each grade	Positive (%)	86 (90.5)			222 (92.9)			198 (88.4)		506
	Negative (%)	7 (7.4)			13 (5.4)			26 (11.6)		46
	Neutral (%)	2 (2.1)			4 (1.7)			0		6

	Total (%)	95 (100)			239 (100)			224 (100)		558

**Table 4:** Perceived program effectiveness across study 1 to study 8.

Ecological level	Subdomains	Descriptions	S1			S2			S3		Total
			Study 1 2005/06 EIP	Study 2 2006/07 FIP	Study 3 2008/09 FIP	Study 4 2006/07 EIP	Study 5 2007/08 FIP	Study 6 2008/09 FIP	Study 7 2007/08 EIP	Study 8 2008/09 FIP	
Societal	Social norms	Fostered prosocial norms or involvement	3	26	2	7	9	8	0	7	62
	Social responsibility and affairs	Enhanced knowledge about China and Hong Kong	6	9	0	1	0	0	0	0	16
		Enhanced knowledge about public affairs	1	0	0	0	0	0	0	0	1
		Recognized citizen's responsibilities	4	10	0	3	0	0	0	0	17

		Subtotal	14	45	2	11	9	8	0	7	96

School	School belongingness	Increased belongingness to school	0	0	0	0	0	0	1	0	1

		Subtotal	0	0	0	0	0	0	1	0	1

Familial	Family relationships	Enhanced relationships with parents and other family members	5	22	19	12	10	4	1	11	84

		Subtotal	5	22	19	12	10	4	1	11	84

Interpersonal	General interpersonal competence	Enhanced team spirit	9	15	0	18	0	0	0	0	42
		Made friends	7	15	0	6	0	0	0	0	28
		Got along with others	19	65	0	41	0	0	0	0	125
		Enhanced peer relationship	0	28	11	0	23	12	12	17	103
		Enhanced relationship with instructors/social workers	0	2	0	0	4	5	4	8	23
		Able to differentiate between healthy and unhealthy friends	7	22	0	2	4	0	0	0	35
		Able to handle love affairs	0	0	0	4	0	0	0	0	4
		Strengthened social bonding	0	0	0	22	0	0	8	0	30
		Enhanced social competence	0	17	0	21	0	0	0	0	38

		Total in subcategory	42	164	11	114	31	17	24	25	428

	Specific interpersonal competence	Improved interpersonal skills	0	0	14	0	8	7	0	19	48
		Improved communication skills	3	30	8	21	16	15	9	8	110
		Enhanced presentation ability	0	0	0	15	0	0	0	0	15
		Learned to accept, care, trust, respect, and understand others	3	38	15	19	11	7	13	18	124
		Promoted cooperation with others	0	4	4	0	19	1	10	4	42
		Increased willingness to express	0	0	0	0	3	0	3	0	6

		Total in subcategory	6	72	41	55	57	30	35	49	345

		Subtotal	48	236	52	169	88	47	59	74	773

Personal	General gains	Promoted students' development	0	0	2	0	32	4	35	10	83
		Acquired skills/knowledge	14	26	6	31	28	9	0	29	143
		Learned something useful and applicable to daily life	13	18	0	0	0	0	0	4	35
		Helpful to students	0	0	13	0	4	8	0	17	42
		Positive changes on students	0	0	0	0	0	0	0	9	9

		Total in subcategory	27	44	21	31	64	21	35	69	312

	Behavioral competence	Improved refusal skills	0	11	0	0	0	0	0	0	11
		Improved decision-making skills	0	0	0	0	0	7	0	8	15
		Learned financial management skills	0	0	0	0	0	0	5	3	8
		Enhanced time management skills	0	0	0	0	1	0	2	0	3
		Enhanced leadership skills	0	0	0	0	0	0	1	0	1
		General behavioral competence	0	0	2	0	15	3	8	4	32

		Total in subcategory	0	11	2	0	16	10	16	15	70

Personal	Cognitive competence	Promoted rational and analytical thinking	5	18	0	26	0	0	0	0	49
		Enhanced creativity	0	2	0	0	2	0	0	0	4
		Improved problem-solving skills	7	26	13	22	14	7	9	15	113
		General cognitive competence	0	0	4	6	22	5	13	20	70

		Total in subcategory	12	46	17	54	38	12	22	35	236

	Moral competence	Cultivated personal ethics/virtues	2	6	6	25	0	3	0	18	60
		Enhanced responsibility	0	0	0	7	0	0	0	0	7
		Enhanced empathy	0	3	0	6	0	0	0	0	9
		Promoted the idea of equality and fairness	3	0	0	0	0	0	0	0	3
		Able to distinguish between right and wrong	5	54	3	19	0	3	0	7	91
		Able to correct bad habits/defects	4	14	0	3	0	0	0	0	21
		Cultivated proper attitudes towards sex and love relationship	0	0	0	0	4	0	4	10	18
		General moral competence	0	0	0	0	4	0	15	6	25

		Total in subcategory	14	77	9	60	8	6	19	41	234

	Emotional competence	Enhanced emotional management/expression	11	57	27	28	9	9	10	17	168

		Total in subcategory	11	57	27	28	9	9	10	17	168

	Beliefs in the future	Promoted beliefs in the future	0	0	0	0	0	1	5	12	18
		Assistance in career planning	0	0	0	0	0	0	0	4	4
		Facilitated goal setting	6	23	4	29	5	4	9	9	89

		Total in subcategory	6	23	4	29	5	5	14	25	111

	Resilience	Cultivated resilience	0	0	4	24	13	15	10	20	86
		Optimism	1	5	0	9	0	4	0	0	19
		Ways to face adversity	0	12	0	0	0	0	0	0	12

		Total in subcategory	1	17	4	33	13	19	10	20	117

	Positive self	Facilitated personal growth	11	42	0	34	0	0	0	0	87
		Developed clear and positive identity	0	0	0	0	0	1	0	1	2
		Enhanced self-efficacy	0	0	4	2	3	3	0	5	17
		Enhanced self-confidence	3	27	0	9	2	3	14	9	67
		Enhanced self-determination	0	14	0	15	2	1	4	2	38
		Facilitated self-understanding	18	41	7	28	18	5	30	13	160
		Developed potentials/widened horizon	0	0	0	0	0	0	0	7	7

		Total in subcategory	32	124	11	88	25	13	48	37	378

	Cherishing life	Learned to cherish life	1	3	0	41	9	8	2	13	77
		Cultivated positive life views/learned life principles	0	0	3	0	0	14	0	39	56
		Learned to treasure people and things	1	21	0	12	0	0	9	0	43
		Self-reflection	3	9	7	9	3	3	11	18	63
		Promoted reflection of life	5	5	0	28	12	13	0	8	71

		Total in subcategory	10	38	10	90	24	38	22	78	310

		Subtotal	113	437	105	413	202	133	196	337	1936

Others	Positive comments	Interest in learning	1	4	0	10	2	0	0	0	17
		Stimulating	2	0	0	0	0	0	0	0	2
		Fruitful	10	0	0	0	0	0	0	0	10
		Academic improvements	0	0	0	0	0	0	0	4	4
		Healthy living style	0	0	0	12	2	0	0	0	14
		Others	10	8	4	8	0	6	0	4	40

		Total in subcategory	23	12	4	30	4	6	0	8	87

	Negative comments	Program was not effective	0	0	1	0	0	2	0	0	3

		Total in subcategory	0	0	1	0	0	2	0	0	3

Others	Neutral comments	Could not help much on students' development	0	0	0	0	0	0	3	0	3
		Others	0	0	0	0	0	0	0	3	3

		Total in subcategory	0	0	0	0	0	0	3	3	6

		Subtotal	23	12	5	30	4	8	3	11	96

Grand total			203	752	183	635	313	200	260	440	2986
